# An Approach for Data Mining of Electronic Health Record Data for Suicide Risk Management: Database Analysis for Clinical Decision Support

**DOI:** 10.2196/mental.9766

**Published:** 2019-05-07

**Authors:** Sofian Berrouiguet, Romain Billot, Mark Erik Larsen, Jorge Lopez-Castroman, Isabelle Jaussent, Michel Walter, Philippe Lenca, Enrique Baca-García, Philippe Courtet

**Affiliations:** 1 Adult Psychiatry Brest Medical University Hospital at Bohars Brest France; 2 EA 7479 Soins Primaires, Santé Publique, Registre des Cancers de Bretagne Occidentale Université de Bretagne Occidentale Brest France; 3 Mental Health Department University Hospital of Brest Brest France; 4 F-29238 Laboratoire des Sciences et Techniques de l'information de la Communication et de la Connaissance IMT Atlantique Brest France; 5 Black Dog Institute University of New South Wales Sydney Australia; 6 Inserm U1061 Crisis Admission Center University Hospital of Nîmes Nîmes France; 7 Inserm U1061 La Colombières Hospital University of Montpellier Montpellier France; 8 Carlos III Institute Of Health Centro de Investigation en Salud Mental Madrid Spain; 9 Department of Psychiatry Universitad Catolica del Maule Talca Chile; 10 Department of Psychiatry General Hospital of Villaba Madrid Spain; 11 Department of Psychiatry University Hospital Rey Juan Carlos Mostoles Spain; 12 Department of Psychiatry University Hospital Infanta Elena Valdemoro Spain; 13 Psychiatry Department Universidad Autónoma de Madrid Madrid Spain; 14 Department of Psychiatry Instituto de Investigación Sanitaria de la Fundación Jiménez Díaz Madrid Spain; 15 Department of Emergency Psychiatry and Acute Care Centre Hospitalier Universitaire de Montpellier Université Montpellier Montpellier France; 16 Fondamental Foundation Créteil France

**Keywords:** clinical decision support system, data mining, electronic health, mobile phone, prevention, suicide, suicide attempts

## Abstract

**Background:**

In an electronic health context, combining traditional structured clinical assessment methods and routine electronic health–based data capture may be a reliable method to build a dynamic clinical decision-support system (CDSS) for suicide prevention.

**Objective:**

The aim of this study was to describe the data mining module of a Web-based CDSS and to identify suicide repetition risk in a sample of suicide attempters.

**Methods:**

We analyzed a database of 2802 suicide attempters. Clustering methods were used to identify groups of similar patients, and regression trees were applied to estimate the number of suicide attempts among these patients.

**Results:**

We identified 3 groups of patients using clustering methods. In addition, relevant risk factors explaining the number of suicide attempts were highlighted by regression trees.

**Conclusions:**

Data mining techniques can help to identify different groups of patients at risk of suicide reattempt. The findings of this study can be combined with Web-based and smartphone-based data to improve dynamic decision making for clinicians.

## Introduction

### Suicide Risk Assessment

Over 800,000 people die of suicide every year, and it is estimated that for each suicide, there may have been >20 other attempted suicides. A previous attempt is the major predictor of death by suicide [1]. However, many other outcomes associated with suicidal behaviors should be considered in the preventive and therapeutic decision-making process for effective prevention [2]. Thus, clinical [3], environmental [4], and genetic [2] suicide risk factors have been intensively studied among suicide attempters. Indeed, attempters provide data to identify suicide-related risk factors, and such at-risk patients are a privileged target for proper prevention and intervention strategies (eg, by mitigating risk factors or by maintaining contact with clinical support) [5]. Empirically informed suicide risk assessment frameworks are useful in guiding the evaluation and treatment of individuals presenting with suicidal symptoms. Actual guidelines recommend the systematic identification of risk factors based on risk assessment scales [6]. Nevertheless, the limits of actual risk assessment procedures may be a false reassurance for clinicians, and the conflation of risk assessment and risk prediction may be confusing to clinicians [7]. Therefore, there is an urgent need for an innovative tool that could integrate both empirical and structured assessment to support decision making in suicide prevention.

### Clinical Decision-Support Systems

Decision-support tools help providers in their decision-making process. The use of these tools has been on the rise in recent years owing to their ability to bring evidence-based medicine to the point of care. A clinical decision-support system (CDSS) is a health information system that is integrated into electronic health records (EHR), enabling easy and effective use by physicians [8]. The CDSS incorporates individual patient data, a rule engine, and a medical knowledge base to produce a patient-specific assessment or recommendation of a management plan [9]. The CDSS usually relies on the processing of clinical data gathered into EHRs. However, these techniques have been poorly explored in mental health and the suicide-prevention setting [10].

Thus, there is still an important need to develop a CDSS that supports clinician decision makers to choose, for example, the most appropriate treatment, the nature of a psychosocial strategy, or the duration of treatment in suicide prevention strategies. A key feature of such a CDSS is to identify a patient’s risk in terms of a repeated attempt, the number of reattempts, or suicide death within a period of time. The development of both passive and active collection of patients’ data provides the opportunity to improve clinician knowledge and thus determine risk factors and relevant combinations of risk factors [11].

### Aims

This study aims to combine data from EHRs to provide support to decision making for clinicians in suicide prevention. We present the main results of a data mining process on a sample of suicide attempters to first identify groups of similar patients and then identify risk factors associated with the number of suicide attempts. We hypothesize that a data mining process helps to better characterize the population of suicide attempters by identifying the most relevant groups of patients and their associated risk factors for suicide reattempt (or other variables of interest). The ultimate goal is to build a CDSS for clinician decision support and propose a personalized prevention and intervention strategy to each patient.

## Methods

### Patient Recruitment

Suicide attempters aged >18 years were recruited from consecutive admissions to the Emergency Department or specialized Acute Care Unit of three university hospitals (University Hospital Ramon y Cajal, Madrid, Spain; Fundación Jimenez Diaz, Madrid, Spain; and Academic Hospital of Montpellier, Montpellier, France) between 1994 and 2006. Owing to their specific characteristics [12,13], major suicide repeaters were excluded. One of the hospitals is part of the Spanish National Health System and the other, the French National Health System; both hospitals provide medical coverage for all emergencies in a catchment area covering a population of around 500,000 people in Madrid and 400,000 in Montpellier. After providing a complete description of the study to participants, written informed consent was obtained. Trained psychiatrists or psychologists interviewed all patients before discharge. The study was approved by the local research ethics committees in Madrid and Montpellier (CPP Montpellier Sud-Méditerranée IV, CHU Montpellier). The research followed the Code of Ethics of the World Medical Association (Declaration of Helsinki). Protocols and assessment procedures in both centers are based on the Columbia Suicide History Form [14].

### Procedure and Clinical Assessment

The French or Spanish version of the Mini-International Neuropsychiatric Interview (MINI) [15] was used to obtain Axis I Diagnostic and Statistical Manual of Mental Disorders - 4th edition diagnoses. Psychiatric diagnoses were classified in the following categories: mood disorder (specifying depression or bipolar disorder), anxiety disorders, obsessive-compulsive disorder, alcohol or drug misuse, psychotic disorders, eating disorders, somatoform disorders, and adjustment disorders. The lifetime diagnosis was determined using a best-estimate procedure. The psychiatrist in charge of the patient’s care assigned the diagnosis based on MINI interviews, medical records, and information from relatives, when available.

Suicide risk was assessed using the Suicide Intent Scale [16], a semistructured 15-item rating scale yielding a global score that indicates the severity of suicidal intent. The Risk-Rescue Rating Scale [17] is a 10-item interviewer-administered scale designed to assess the lethality and intent of a suicide attempt, measuring the life risk derived from it, and the likelihood of a rescue intervention at the time of the attempt.

### Statistical Methods

A robust data-qualification process was performed to ensure data quality and consistency before statistical analyses. Although data were intended to be collected according to the same clinical procedures, quality variations were expected between the hospitals. Missing data were identified, and a variable was retained only when the completion rate reached 70%. When needed, new variables were created. For instance, 34 answers of the Barratt Impulsiveness Scale survey, version 10 (BIS10), were treated to build 3 scores of impulsiveness in terms of motor impulsivity, attentional impulsivity, or nonplanning impulsivity [18]. For each question of the BIS10, the score ranged from 1 (low impulsivity) to 4 (high impulsivity). The total score ranges from 34 to 136 points. The subscores ranged from 11 to 44 points for motor and attentional impulsivity and from 12 to 48 points for nonplanning impulsivity.

Unidimensional and two-dimensional analyses for both quantitative and qualitative variables were carried out. In addition, Fisher-Snedecor procedures were used to compare the two subgroups (male vs female) when needed. An unsupervised approach was used to extract homogeneous patterns from the data without any prior hypothesis. The approach is based on a multiple correspondence analysis (MCA) of qualitative variables to reduce the dimensionality. It consists of representing patients in a factorial space where each dimension is a combination of initial variables. Quantitative variables (eg, age) are not used during the calculations but are projected onto the factorial space. Hierarchical Clustering on Principal Components is then performed from the patients’ representation in the initial factorial space. Hierarchical clustering has many advantages, including the construction of a hierarchical tree called dendrogram that enables a visual interpretation of the dataset. The dendrogram depicts the emergence of groups of patients who share common risk patterns. In addition, it facilitates discussion between statisticians and practitioners to choose the optimal number of clusters. Each cluster was then interpreted through the association between the cluster and the list of qualitative and quantitative variables (V test). In the second step, the focus was on the variable of interest—the number of suicide attempts. Recursive partitioning has been used as a multivariable procedure that classifies individuals (patients) by successively splitting into subpopulations. Furthermore, a regression tree was built, and the number of suicide attempts was explained by different binary tests on predictive variables.

## Results

### Population Description

From the original database, the first step relied on data qualification. Several redundancies (eg, duplicated surveys or alternative coding) were observed among 263 initial variables. Subsequently, a completion threshold was applied to the resulting variables, and only 23 variables satisfied a 70% minimum completion rate. Three additional variables related to the types of impulsivity (as described above) were added. With respect to the 2802 initial patients, we decided to keep only suicide attempters with a 100% completion rate for the 26 variables. In the final filtering, 5 variables were disregarded for redundancy or useless purpose (the type of patients, assessment date, source, and year and day of birth). This rigorous process ensured high data quality for both patients and variables; it also provided a final dataset of 681 patients and 21 variables. Participants were predominantly young (mean age 40.1 years), female, employed, and married. Most patients included in the final analysis had a history of mental disorders, including major depression (482/681, 70.8%), bipolar disorder (160/681, 23.0%), dysthymic disorder (30/681, 4.4%), obsessive-compulsive disorder (58/681, 8.5%), and alcohol misuse (178/681, 26.1%) (Table 1).

**Table 1:** Clinicosociological main features of the postfiltering dataset of 681 suicide attempters.

Features				Value
**General features**				
	**Qualitative variables, n (%)**			
		**Sex**		
			Female	493 (72.4)
			Male	188 (27.6)
		**Marital status**		
			Single	239 (35.1)
			Married	240 (35.2)
			Separated or divorced	181 (26.6)
			Widowed	21 (3.1)
		**Children**		
			No	272 (39.9)
			Yes	409 (60.1)
		**Education**		
			Low	31 (4.6)
			Intermediate	368 (54.0)
			High	282 (41.4)
		**Employment**		
			Employed	451 (66.2)
			Unemployed	110 (16.2)
			Incapacity	41 (6.0)
			Retired	79 (11.6)
	Quantitative variable, age (years), median (Q1-Q3)			40.6 (28-49.6)
**Clinical features**				
	**Qualitative variables, n (%)**			
		**History of mental disorder**		
			No	6 (0.9)
			Yes	675 (99.1)
		**History of family suicidal behavior**		
			No	424 (62.3)
			Yes	257 (37.7)
		**Lifetime major depression**		
			No	199 (29.2)
			Yes	482 (70.8)
		**Lifetime bipolar disorder**		
			No	521 (76.5)
			Yes	160 (23.5)
		**Lifetime dysthymic disorder**		
			No	651 (95.6)
			Yes	30 (4.4)
		**Lifetime obsessive-compulsive disorder**		
			No	623 (91.5)
			Yes	58 (8.5)
		**Lifetime eating disorder**		
			No	571 (83.8)
			Yes	110 (16.2)
		**Lifetime alcohol-drug misuse**		
			No	465 (68.3)
			Yes	216 (31.7)
		**Lifetime substance misuse**		
			No	586 (86.0)
			Yes	95 (14.0)
		**Lifetime alcohol abuse**		
			No	503 (73.9)
			Yes	178 (26.1)
	**Quantitative variable**			
		Number of suicide attempts, median (Q1-Q3)		2 (1-3)
		**Barratt Impulsiveness Scale survey - version 10, scores (range)**		
			Motor impulsivity	26 (22-30)
			Attentional impulsivity	27 (23-30)
			Nonplanning impulsivity	28 (24-31)

### Principal Outcome: Clustering of Patients

**Figure 1:**
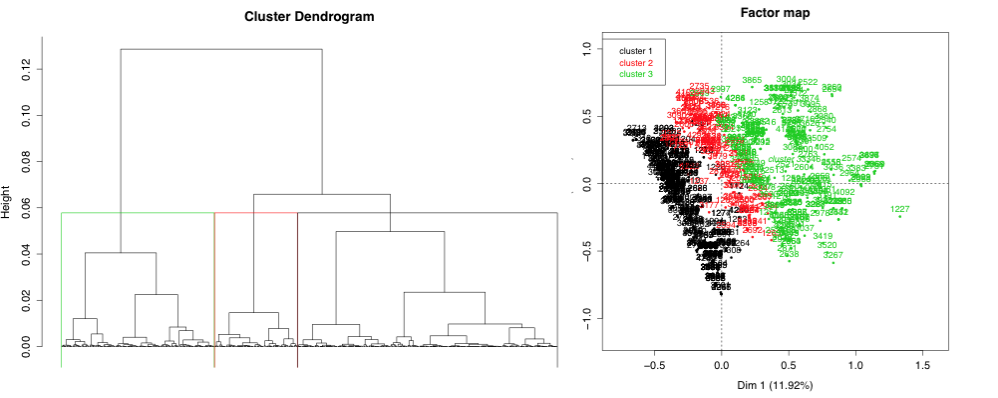
Hierarchical clustering (left) and multiple correspondence analysis factor map (right) with three projected clusters.

The first step of the analysis was to perform an MCA to reduce the dimension, followed by a hierarchical clustering from the principal components to highlight groups of homogeneous patients. The tree structure (in terms of inertia gain) and a discussion between statisticians and practitioners allowed us to study patients partitioned into three clusters. Figure 1 shows the cluster dendrogram and the projection of the three clusters onto the factor map; the factor map represents a two-dimensional projection of the first two dimensions only.

We conducted an in-depth analysis of the 3 groups for data interpretation. Statistical association tests (V tests) enabled identification of over- or underrepresented modalities in the three clusters. Cluster 1 was mainly related to an average patient profile of women (positive association V test, *P*<.001) who did not misuse drugs, substances, or alcohol (*P*<.001) and without bipolar disorder (*P*<.001), but with previous or current episodes of depression (*P*<.001) and other mental health disorders (*P*=.01). Cluster 3 was, in contrast, associated with men (*P*<.001) and drug, substance, and alcohol misuse (*P*<.001); in this group, patients were mainly single (*P*=.01), with no children (*P*=.006) and no experience of depression (*P=*.006). Compared with clusters 1 and 3, cluster 2 was neutral in terms of gender, but this group was related to people with a work incapacity (*P*=.03), low education level (*P*=.02), possible bipolar disorder (*P*<.001), and no drugs or alcohol misuse (*P*<.001) and without episodes of depression or other mental health disorders (*P*<.001). Thus, without any prior hypothesis, this unsupervised approach underlined three homogeneous groups. Gender appeared as a crucial marker for two of the three groups.

### Secondary Outcome: Identification of Factors Associated With the Risk of Repeated Suicide Attempts

**Figure 2:**
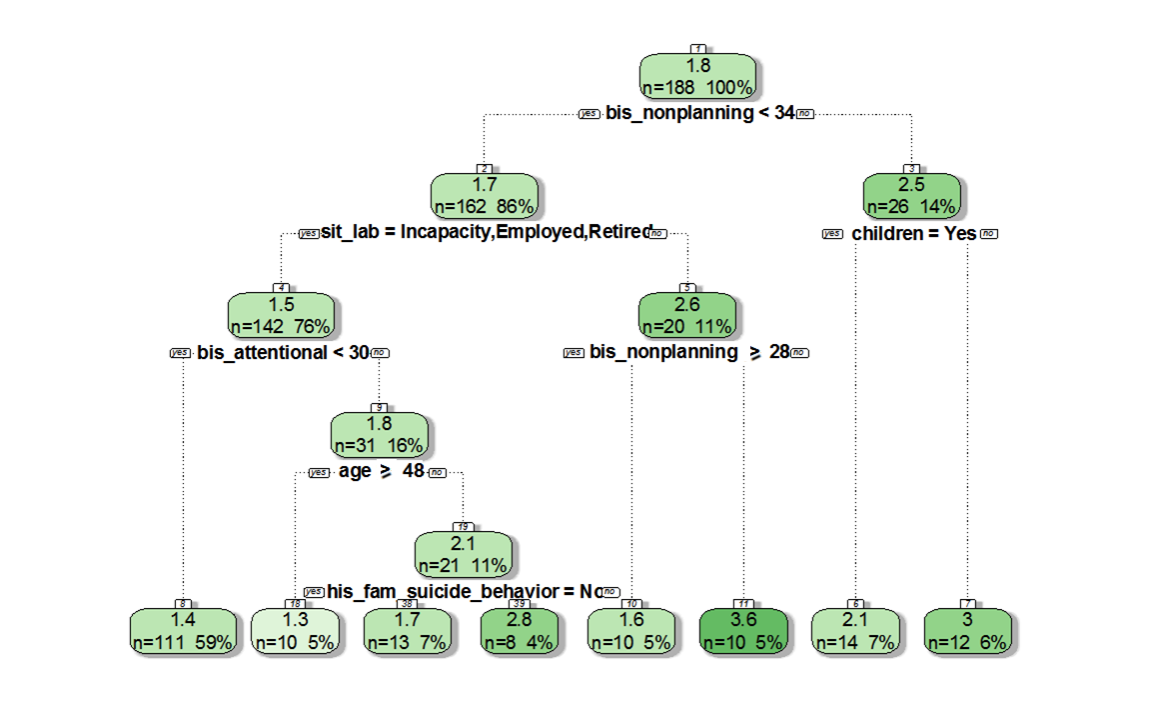
The decision tree on the variable "number of suicide attempts" according to gender "male". BIS: Barratt Impulsiveness Scale.

**Figure 3:**
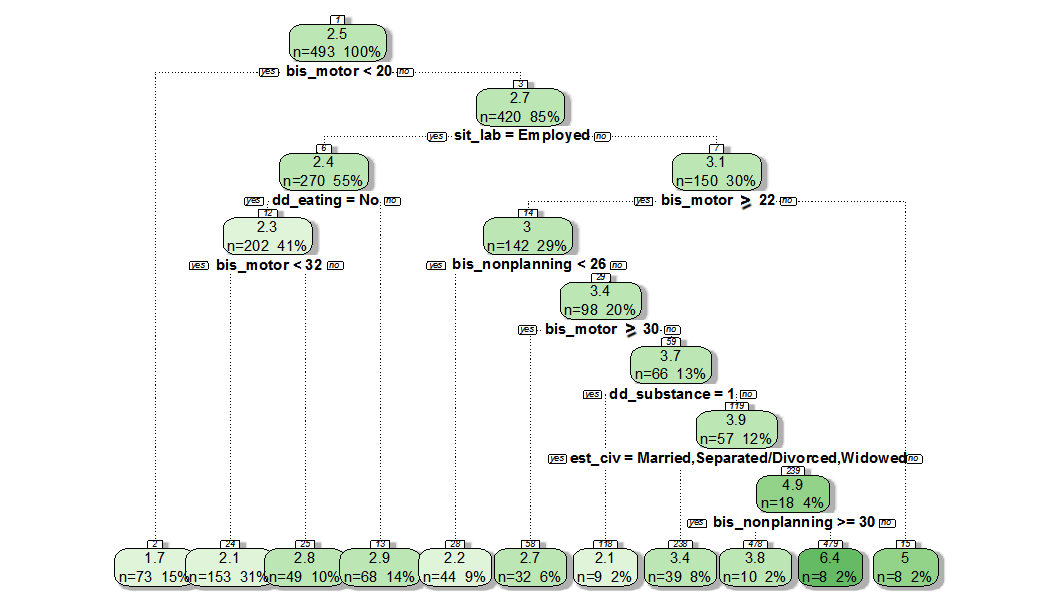
The decision tree on the variable “number of suicide attempts” according to gender “female”. BIS: Barratt Impulsiveness Scale.

The second step of the analysis aimed to identify factors associated with a higher risk of suicide attempts (variable “number of suicide attempts”) for men and women separately, following the principal outcome. Figure 2 depicts the regression tree for male patients, while Figure 3 shows the results for female patients only. For both groups, we noted that impulsivity aspects (including the motor section, nonplanning section, and attentional impulsivity section of the BIS interview) were relevant factors explaining the number of suicide attempts for a patient. Furthermore, higher scores were associated with a higher number of attempts for patients.

While analyzing both groups, the first conclusion is a clear difference between genders. For instance, eating disorders are linked to a higher number of suicide attempts for women (mean 2.9 in women vs 2.3 in men, *P*=.005), while a history of familial suicidal behavior (mean 2.8 in men vs 1.7 in women, *P*<.001) and the employment status are risk factors for some men. In particular, unemployed men with higher scores at the BIS interview were at higher risk of suicide repetition. Older age and having children were also identified as risks factors.

## Discussion

### Principal Findings and Integration into a Dynamic Clinical Decision-Support System

A systematic assessment before discharge from hospital has allowed building of a large database suitable for modern data mining techniques. In this study, we identified clusters of suicide attempters and variables that may explain the repetition of suicide attempt in suicide attempters. This study shows how a simple structuration of the assessment of discharged patients after a suicide attempt may provide relevant data for clustering methods. The clustering may help clinicians allocate a patient into a risk cluster. Therefore, it is the first step of the CDSS design. This model may lead to a stratified approach in decision making for suicide prevention. Furthermore, analyzing larger datasets could allow the discovery of new risk factors that are not currently considered relevant during clinical interviews. However, we did not propose a model for suicide prediction; our model mining big databases is a prerequisite toward better decision making for suicide prevention. Furthermore, this model could also be applied to other data sources like personal health records or ecological momentary assessment (EMA).

Our findings are in line with recent studies showing how suicide risk assessment could lead to patient clustering from a preventative perspective [19]. Eleven clinically relevant items related to the characteristics of suicidal behavior were submitted to a Hierarchical Ascendant Classification; the results showed that most individuals were included in a cluster characterized by less lethal means and planning (“impulse-ambivalent”). The second cluster featured more carefully planned attempts (“well-planned”), more alcohol or drug use before the attempt, and more precautions to avoid interruptions. Finally, the third cluster included individuals reporting more attempts (“frequent”), more often serious or violent attempts, and an earlier age at the first attempt. In addition, differences across clusters by demographic and clinical characteristics were found, particularly with the third cluster whose participants had experienced high levels of childhood abuse. Overall, a systematic, structured assessment may help clinicians characterize suicide risk better and personalize prevention strategies. We believe that electronic health and data mining techniques may help us to reach this goal.

In this study, participants were assessed by trained clinicians before discharge from the ED. Data were captured using paper-based formularies of the actual MeMind Web-based EHR [20]; these data could have been captured by our MeMind Web app [20] designed to gather observational data through an EHR interface and perform EMA [18,19]. This Web-based software has two distinct views—the EHR view (for clinicians) and the EMA view (for patients). The “EHR” view is designed to be used by doctors and nurses during the face-to-face assessment. As current EHRs, the app also collects sociodemographic, diagnostic, and pharmacological treatment information within the “assessing suicide” protocol. Sociodemographic variables included age (defined as the age at the index episode), sex, profession, current working status, marital status, number of children (if any), and educational level. Moreover, family history of suicidal behavior, age at the first suicide attempt, and violence of the suicide attempt were measured. The EMA view for patients to track their symptoms was not used in this study. Clinicians and patients can access the Web app either from a computer or their personal mobile phone. EMA involves repeated sampling of subjects’ behaviors and experiences in real time in their natural environment. EMA has been successfully used for real-time self-reporting of symptoms and behavior. For example, Husky et al showed the utility and feasibility of using EMA to study suicidal ideation [4]. For this study, patients did not have any access to this interface. Such a Web app may represent the future of suicide risk assessment, as it allows data mining of static data (EHR data provided by a single clinical assessment) and dynamic data (smartphone data provided by EMA techniques); these data can be processed to build a dynamic CDSS [19].

### Limitations

#### Focus on Suicide Attempters

In this study, patients were recruited after a suicide attempt. We postulate that the development of a CDSS would be more relevant in a population of suicide attempters. Suicide attempters are also defined as an “indicated population” [2] who warrant the maximal attention of health care service owing to the risk of reattempt. Treatment and follow-up strategies are well described in the guidelines for suicide attempters. Therefore, we were able to propose treatment to each participant based on actual recommendations. However, applying these strategies to samples of patients with suicide ideation but without a history of suicide attempt would be hypothetical and ethically controversial. Owing to the epidemiological specificities of both populations, a specific model would have to be built for each population. In terms of multiple suicide attempters, a relevant perspective is to focus on the temporality of suicide attempts rather than the absolute number; to achieve this objective, finer data are needed.

#### Missing Data: The Data Mining Challenge

This study illustrates the need for high-quality and large databases for extracting significant patient profiles or risk factors. In this study, starting from an initial set of 2802 patients with 263 variables, the data-qualification process resulted in a final sample of 681 patients with 21 complete variables. Although this volume of data already ensures statistical significance, it underlines the importance of better ways to standardize data collection in participating institutions. The CDSS quality strongly depends on input data. Moreover, a critical challenge may be the clinician acceptance of such tools that directly impact the completion rate of the EHR [21]. Another option could be the integration of other data sources, such as personal health records and EMA [21]. Overall, the use of larger databases will refine different profiles of patients and dynamically improve the personalized prevention strategies, thanks to the EMA data.

### Recommendations for Suicide Prevention

#### From Guidelines to Clinical Decision-Support Systems in Suicide Prevention

Guidelines recommend that all patients presenting to the hospital services with self-harm should receive a psychological assessment before discharge, to determine the risk of further reattempt [6]. This assessment should also help clinicians choose the most appropriate treatment while considering clinical guidelines and patient-specific risks factor. However, reviews have addressed the challenge clinicians working in the emergency setting face when they rely on these tools to perform decision making [7]. As EHRs are extensively used in emergency services and psychiatric departments, we propose integrating CDSS features regarding an individual’s risks factors into EHRs.

#### Toward Dynamic Clinical Decision-Support Systems

Most clinicians have use EHRs daily in emergency services and psychiatric units. However, few institutions have taken advantage of recent technological advances opportunities in risk assessment. Combining electronic health–based assessment with data mining techniques represents an opportunity to foster suicide-prevention research. This new paradigm is useful in providing personalized intervention strategies by itself, but it also affords the opportunity to identify novel mechanisms to be targeted in suicide-prevention strategies. In addition, we believe that computational models can provide data-assisted ideas emerging from these repositories and will have special appeal for the empirically minded clinicians [7].

Although studies have highlighted the value of self-reports in clinical assessment, they are rarely routinely implemented [22]. Internet and mobile technologies are ideal for self-monitoring assessment and ecological observational studies. Mobile phones are generally kept on at all times and carried everywhere, making them an ideal platform for the broad implementation of EMA technology. For example, Husky et al conducted a study providing support for the use of EMA in the identification of suicidal ideation in outpatients [23]. These techniques may help clinicians identify risky events occurring during follow-up. Overall, these EMA data could also be mined and integrated into the decision-making process.

### Conclusions

**Figure 4:**
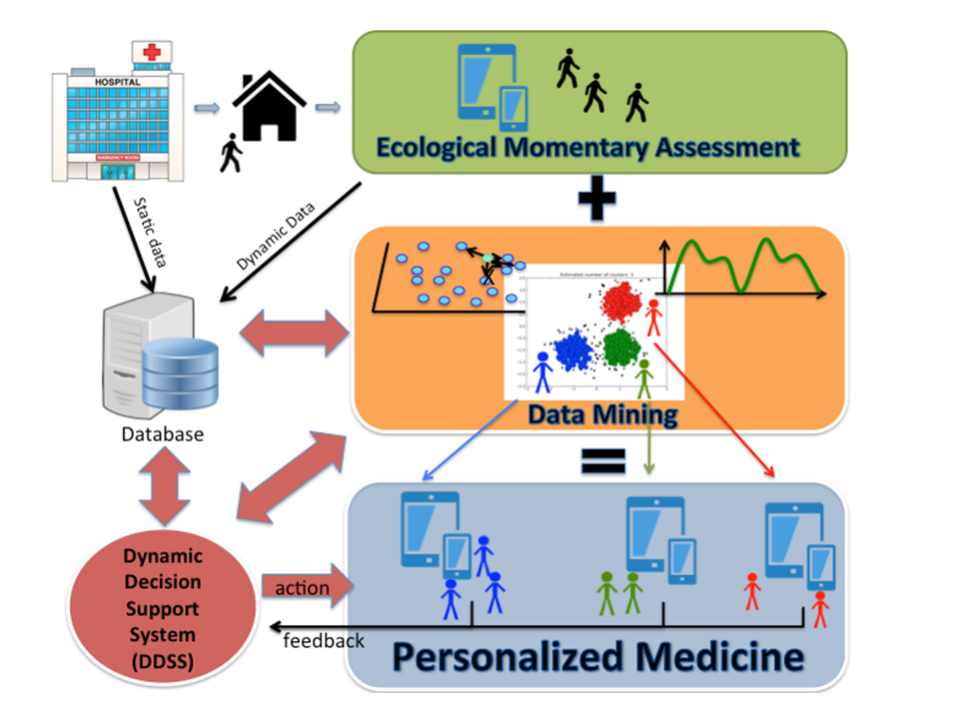
The decision-support system based on ecological momentary assessment and data mining.

The next step is to take advantage of new technologies and current developments of Web-based mobile apps to design the next-generation dynamic CDSS (Figure 4). As emerging mobile health (mHealth) techniques in suicide prevention strategies also produce relevant data, this study proposes a new model of the decision-support system based on the data mining proceedings from face-to-face assessment and mHealth EMA. Our strategy relies on the processing of static data (initial assessment) and dynamic data (EMA) able to instantaneously deliver to clinicians decision support regarding a specific patient. Indeed, mHealth apps allow patients to report their physical or mental health conditions and symptoms, hence providing dynamic data able to enrich studies, confirming or rejecting statistical hypotheses [24]. Such dynamic data based on EMA will form the core of a dynamic decision-support system, which will adapt its recommendations to patients’ characteristics (Figure 4).

## Abbreviations

CDSS: clinical decision-support system
EHR: electronic health records
EMA: ecological momentary assessment
MCA: multiple correspondence analysis
mHealth: mobile health
MINI: Mini-International Neuropsychiatric Interview
